# Mechanisms of Nanophase-Induced Desorption in LDI-MS. A Short Review

**DOI:** 10.3390/nano7040075

**Published:** 2017-04-02

**Authors:** Rosaria Anna Picca, Cosima Damiana Calvano, Nicola Cioffi, Francesco Palmisano

**Affiliations:** Dipartimento di Chimica, Università Degli Studi di Bari “Aldo Moro”, via E. Orabona 4, Bari (BA) 70126, Italy; rosaria.picca@uniba.it (R.A.P.); cosimadamiana.calvano@uniba.it (C.D.C.); francesco.palmisano@uniba.it (F.P.)

**Keywords:** SALDI, nanomaterial, ion desorption efficiency, internal energy transfer, benzylpyridinium, laser-nanoparticle interaction

## Abstract

Nanomaterials are frequently used in laser desorption ionization mass spectrometry (LDI-MS) as DI enhancers, providing excellent figures of merit for the analysis of low molecular weight organic molecules. In recent years, literature on this topic has benefited from several studies assessing the fundamental aspects of the ion desorption efficiency and the internal energy transfer, in the case of model analytes. Several different parameters have been investigated, including the intrinsic chemical and physical properties of the nanophase (chemical composition, thermal conductivity, photo-absorption efficiency, specific heat capacity, phase transition point, explosion threshold, etc.), along with morphological parameters such as the nanophase size, shape, and interparticle distance. Other aspects, such as the composition, roughness and defects of the substrate supporting the LDI-active nanophases, the nanophase binding affinity towards the target analyte, the role of water molecules, have been taken into account as well. Readers interested in nanoparticle based LDI-MS sub-techniques (SALDI-, SELDI-, NALDI- MS) will find here a concise overview of the recent findings in the specialized field of fundamental and mechanistic studies, shading light on the desorption ionization phenomena responsible of the outperforming MS data offered by these techniques.

## 1. Introduction

**Figure 1:**
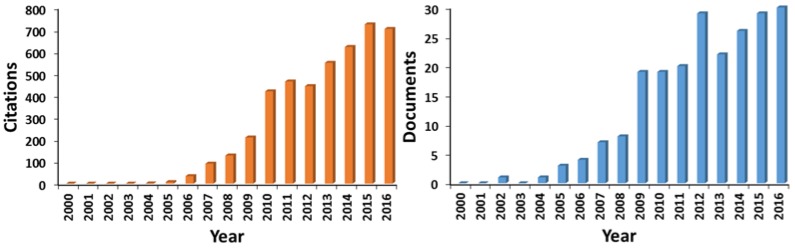
Outcome from Web of Science™ (v. 5.23.2) database searching for “SALDI MS” in topic field (Database accessed on 6 February 2017). The number of citations (**left**) and the number of publications (**right**) are reported per each year in the 2000–2016 timespan.

Nowadays matrix-assisted laser desorption/ionization-mass spectrometry (MALDI-MS) [1] represents a versatile and important technique for the characterization of macromolecules as proteins, DNA, synthetic and bio-polymers [2,3]. Despite its great success, the use of organic matrices introduces several drawbacks in MALDI-MS analysis such as: (i) problematical detection of low molecular weight compounds (to approximately 700 *m/z*), due to background noise of matrix-related ions; (ii) absence of a universal matrix, which implies a preliminary knowledge of the investigated compounds; (iii) difficult quantitative analysis, due to inhomogeneous matrix-analyte co-crystallization. These and related problems encouraged the search for alternative approaches as matrix-free desorption/ionization methods. Surface assisted laser desorption/ionization (SALDI) MS was introduced by Sunner et al. [4], who employed a matrix composed of a suspension of graphite particles in a blend of glycerol, sucrose, and methanol for the analysis of peptides. Such an approach appears closely related to the one originally proposed by Tanaka et al., where a suspension of 30 nm fine cobalt powders in glycerol or water was employed as a matrix for protein analysis [5]. SALDI presented several advantages compared to conventional MALDI such as independence of the excitation laser wavelength over a wide wavelength range, an easy sample preparation and low matrix related background. In fact, materials such as metal oxide nanoparticles or silicon nanowires are scarcely ionized due to their high melting and boiling points, with a resulting limited production of interfering ions in the low mass range. Thanks to these features, SALDI has found extensive application in various fields of interest (e.g., environmental, genomics, forensic, foodomics, and proteomics) as evident from a literature search in a scientific database (Figure 1). Worth of mention are also diverse SALDI variants, such as DIOS (desorption/ionization on silicon) [6], NIMS (nanostructure initiator mass spectrometry) [7], MELDI (material enhanced laser desorption/ionization) [8], SPALDI (silicon nanoparticle assisted laser desorption/ionization) [9], GALDI (graphite assisted laser desorption/ionization) [10], NALDI (nanowire assisted laser desorption/ionization) [11] or nano-PALDI (nanoparticle assisted LDI) [12,13], and AuNPET (cationic gold nanoparticle-enhanced target) [14]. The huge number of different surfaces considered as potential SALDI substrates complicates their classification attempt. A plausible categorization [15], based on their elemental composition, gives rise to three main classes: (i) carbon-based (e.g., pencil graphite [16], activated carbon powders [17], carbon nanotubes [18], graphene and graphene oxide [19,20]); (ii) silicon-based (e.g., silicon nanowires (SiNWs) [21], silicon microcolumn arrays [22], silicon films [23], amorphous silicon [24]); (iii) metal-based (e.g., Ag [25], Pt [26], Au [27,28], TiO_2_ [29], ZnO [30], Pd [31], ZrO_2_, ZrO_2_/SiO_2_ nanorods [32], CdSe quantum dots [33]). However, this classification is somewhat limiting since, for instance, pure carbon can behave as an insulator, a semiconductor or a metal depending on its structure or atomic arrangement. Further, recently explored hybrid systems such as gold nanoparticles (AuNPs) supported on silicon plate with cationic copolymer [34], iron oxide nanoparticles modified with sinapic acid [35] or magnetic NPs coupled to TiO_2_ [36], SiNWs functionalized by Ag nanoparticles [37,38], would not be included as well. Lately, Yonezawa et al. [39] divided SALDI-MS techniques into two categories: (i) nanostructured surfaces such as commercially available DIOS, NALDI, Quickmass, and (ii) nanoparticle systems including diverse inorganic nanoparticles or combination of them. Tarui et al. [34] distinguished “wet” (referring to ultrafine, nanometer- and micrometer-sized, metal particles suspended in glycerol and other liquids) from “dry” SALDI, which includes a large variety of materials such as carbon nanotubes, metal nanoparticles, porous silicon and alumina. Actually, the wet approach is slowly disappearing due to several drawbacks such as severe “sweet spot” phenomena, vacuum deterioration in the ion source, degradation of the instrumental device, reduced ionization efficiency. Law and Larkin [15] proposed some useful criteria to recognize a SALDI method avoiding classification problems: (1) the LDI performance should be superior compared to roughened metal or silicon surfaces; (2) the laser fluence required to attain LDI spectra should be similar to conventional MALDI; (3) the formation of molecular or quasi-molecular ions should be favored as expected for a soft ionization technique; (4) fragmentation, if occurring, should have a predictable and orderly pattern; (5) analysis of a wide range of compounds should be allowed.

The current research in SALDI–MS covers three main topics: (i) engineering novel substrates with improved LDI performance; (ii) developing advanced applications in various fields and (iii) understanding the fundamental SALDI mechanisms. Concerning the first topic, besides the papers already cited, great effort is devoted to fabricate hybrid systems such as α-cyano-4-hydroxycinnamic acid-modified AuNPs [40], immobilized silica and 2,5-dihydroxybenzoic acid on iron oxide magnetic nanoparticles [41], or combination of metal nanoparticles and semiconductor materials [37,38]. It is worth noting the general tendency to move from macroscopically flat surfaces to nanostructured materials, due to several potential benefits, including: an extended dynamic range (due to an increased surface area and analyte loading capacity); improved reproducibility (due to intimate analyte incorporation and deposition homogeneity); superior sensitivity and energy-transfer efficiency (due to the unique optical and thermal properties of nanomaterials).

As for the second topic, SALDI has demonstrated, overall, a valuable tool for the analysis of small molecules [42] in biologically related applications [43]. Currently, many studies are addressed towards the detection of hardly ionizable compounds such as neutral carbohydrates [44,45,46], the improvement of analyte quantification [47], or the development of combined techniques such as infrared spectroscopy bridged to SALDI by means of AuNPs [14] or surface-enhanced Raman scattering spectroscopy (SERS) bridged to SALDI through AgNP functionalized glass fibers [48]. Moreover, nanoparticles and nanostructured substrates have attracted considerable interest in biological applications (e.g., tissue imaging) since they can guarantee more homogeneous deposition methods [49]. In this field, great efforts are aimed to devise new solvent-free approaches (e.g., vapor deposition) to reduce analyte spreading occurring during spray deposition [50].

Contrary, few studies have focused on the physical chemistry of the SALDI process and, therefore, a full understanding of the ion formation mechanisms remains an open issue. The surface morphology, type, form, size, physicochemical properties of the SALDI substrates, the parameters of laser radiation, and the chemical properties of the analyte are critical factors affecting the ultimate analytical performance in terms of ion generation efficiency [51,52,53,54,55]. However, the relative significance of each factor remains ambiguous, as essentially qualitative studies have been reported until now. Definitely, the complete energy transfer pathway is a complex process where several factors influencing desorption and ionization interact each other; moreover, studying desorption and ionization processes taking place in a nanosecond time frame is a tremendous challenge [56].

This paper aims at providing an overview of desorption/ionization mechanisms focusing on specific aspects such as the relationship between ion desorption efficiency and internal energy transfer and other major factors influencing desorption mechanisms from different substrates. Finally, a brief summary on complementary methods, as laser induced breakdown spectroscopy, to study laser radiation-nanophase interactions is also provided, since it could represent a helpful, even indirect, tool to understand SALDI processes.

## 2. Ion-Desorption Efficiencies and Internal Energy Transfer in Laser Desorption/Ionization from Nanostructured Surfaces

A largely empirical approach (of the type “if it works it is good”) is mostly followed in SALDI-MS development. Only few papers have critically investigated the mechanisms behind the interaction of laser radiation with nanomaterials to model LDI phenomena [51,54,57,58,59,60,61]. Confinement effects occur when nanosecond pulsed lasers (typically used in LDI-MS) interact with nanostructures involving nonlinear processes [51]. The behavior of nanostructures of size comparable or smaller than one or more characteristic lengths (e.g., electron and photon mean free paths, excitation diffusion length, near-field range, etc.) defined for a specific material (metal, semiconductor), will be influenced by these peculiar properties. Roughly, energy deposition occurs as the first step when laser beam hits the substrate causing reflection and/or absorption. Reflection can be drastically reduced because of melting and surface plasmon excitations, whereas absorption will depend not only on nanomaterial properties but also on optical properties of adsorbate molecules (coatings, analytes). Thermal processes occur as well, indicating that thermal conductivity (strictly related to size, temperature, surface roughness, and electron thermal conductivity) also plays a role. Obviously, when melting threshold temperature is reached, size and chemistry of nanostructures are drastically modified, causing the occurrence of further processes. In this respect, the majority of the studies dealing with SALDI-MS applications, invoked the thermal-driven desorption (via laser-induced heating) [5,58,60] and heat confinement [48,54,57] as fundamental mechanisms, though other non-thermal mechanisms (e.g., phase transition [51,53,58,62], analyte-NP interactions [51,57,58]) may contribute to the overall LDI process. It is, therefore, often difficult to assess which physicochemical factor plays the most prominent role, as mutual and simultaneous contributions occur [39,42,48,54,57,58]. Chemical composition and size of NPs are certainly key features, as the final physicochemical properties of the nanomaterials will strongly depend on them. To explore how these factors influence SALDI process, the correlation between internal energy transfer and ion desorption efficiency is generally probed.

The simplest approach to study the energetics governing desorption from nanostructured surfaces relies on the use of (preformed) benzylpyridinium (BP) ions as chemical thermometer [63]. Laser desorbed (BP)^+^ ions (*m/z* 170), possessing an amount of internal energy greater than the critical energy of the dissociation reaction, could undergo a simple C–N bond cleavage producing (BP−pyridine)^+^ ions (*m/z* 91). The extent of fragmentation can be evaluated from the relative proportion of the survived intact (BP)^+^ ions, expressed by the survival yield (SY) defined as

SY = (*I*_M_/(*I*_M_ + *I*_F_)) (1)

where *I*_M_ and *I*_F_ indicate the intensity of (BP)^+^ (*m/z* 170), and (BP−pyridine)^+^ (*m/z* 91) ions, respectively [57]. The lower the SY, the higher is the fragmentation degree. The SY provides a simple way to evaluate the extent of internal energy transfer during the laser induced desorption process [4,63] either in SALDI [54,57,58,64] or conventional MALDI [63,65] which, however, can be further complicated by the ionization event(s) in the case of not-preformed ions.

**Figure 2:**
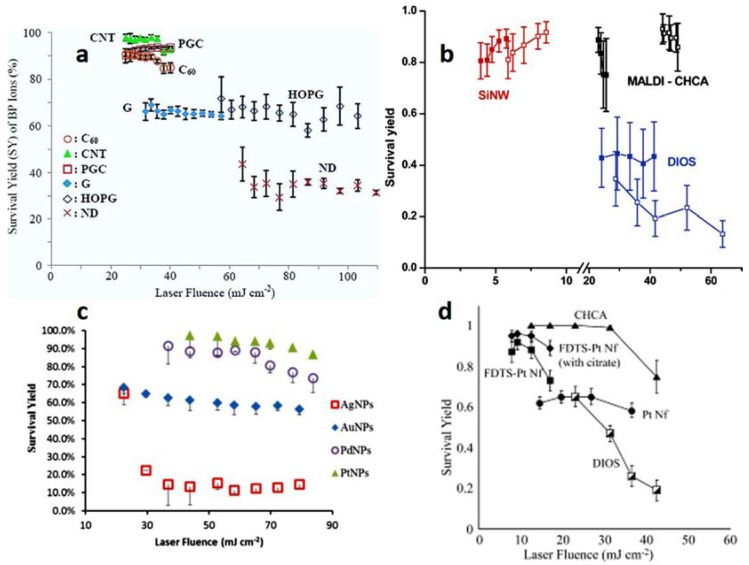
(**a**) Survival yield of BP ions desorbed from various carbon substrates (Buckminsterfullerene = C_60_, Multiwalled Carbon Nanotubes = CNT, nanoporous graphitic carbon = PGC, non-porous graphite particles = G, highly oriented pyrolytic graphite = HOPG, Nanodiamonds = ND) in a range of laser fluencies, adapted with permission from [57]. Copyright American Chemical Society, 2009; (**b**) Survival yields of 4-methyl-BP ions in desorption/ionization from SiNWs, DIOS, and MALDI. All solid symbols are for a N_2_ laser (λ = 337 nm, 4 ns pulse length), and all hollow symbols are for Nd:YAG laser (λ = 355 nm, 22 ps pulse length): (red) perfluorophenyl (PFP)-derivatized SiNW surface, (blue) PFP-modified DIOS, and (black) MALDI from CHCA matrix, adapted with permission from [54]. Copyright American Chemical Society, 2006; (**c**) Survival yield of BP ions desorbed from the noble metal NPs over a range of laser fluence, adapted with permission from [58]. Copyright American Chemical Society, 2015; (**d**) Comparison of molecular ion survival yields of BP in SALDI-MS using Pt nanoflowers (Pt Nfs), perfluorodecyltrichlorosilane (FDTS)-modified Pt Nfs, FDTS-Pt Nfs (with citrate buffer), DIOS, and MALDI with CHCA matrix, adapted with permission from [64]. Copyright John Wiley and Sons, 2010.

It is then possible to evaluate how SY changes as a function of laser fluence. Plots relevant to SY (or SY %) vs. laser fluence for nanomaterials of different composition [54,57,58,64] are presented in Figure 2.

It can be observed that the type of SALDI substrate significantly influences SY. Moreover, the dependence of the SYs on the laser fluence could help in assessing the “softness” (i.e., reduced, or even null, fragmentation) of the LDI process and/or to shed light on desorption mechanisms and underlying influential factors [39,61,65,66,67]. CNTs, C_60_ and PGC exhibited similar high SYs at laser fluencies in the range 20 mJ cm^−2^–40 mJ cm^−2^ (Figure 2a), indicating that poor fragmentation occurs on these substrates [57]. Perfluorophenyl (PFP)-derivatized SiNWs, for instance, showed a fluence threshold (ns laser used) for detectable ion production (4-methylbenzylpyridinium ion as chemical thermometer) considerably lower than the corresponding values for either DIOS or MALDI (see Figure 2b). This can be ascribed to the unique geometry and thermal properties of the SiNWs responsible of thermal energy confinement effects [54]; compared to nanoporous silicon in DIOS, the SYs for desorption from SiNWs are much higher (i.e., SiNWs are much softer than DIOS) [54]. The peculiar geometry of SiNWs can be invoked to explain the differences in plume expansion between DIOS and SiNW-based substrates. A 3D desorbed plume can be assumed for SiNWs, whereas it is considered quasi-one-dimensional for DIOS. In the latter case, plume confinement in DIOS nanopores promotes the energy transfer, which in turn is responsible for reduced SYs. Moreover, the apparent slight increase of SYs with the laser fluence for SiNWs could be artefactual because of the onset of silicon melting and ablation at fluencies above the corresponding threshold [54]. Metal NPs of similar small size (diameters of about 1.7–3.1 nm) display SYs that change slightly with laser fluence (range 20 mJ cm^−2^–80 mJ cm^−2^), and remarkably with their nature (Figure 2c). In fact, Pd and PtNPs can be considered surfaces “softer” than Au and AgNPs [58]. Interestingly, silanized Pt nanoflowers (FDTS-PtNfs) behave quite similarly to SiNWs in terms of laser threshold and ion fragmentation (Figure 2d) [64].

The SY can be used to calculate the ion dissociation rate constant *K*_exp_ that, in turn, is correlated to the internal-energy-dependent rate coefficient curve for (BP)^+^ derived following e.g., the Rice-Ramsperger-Kassel-Marcus (RRKM) formalism [63] (for a detailed discussion see [54,57,58,63]).

**Figure 3:**
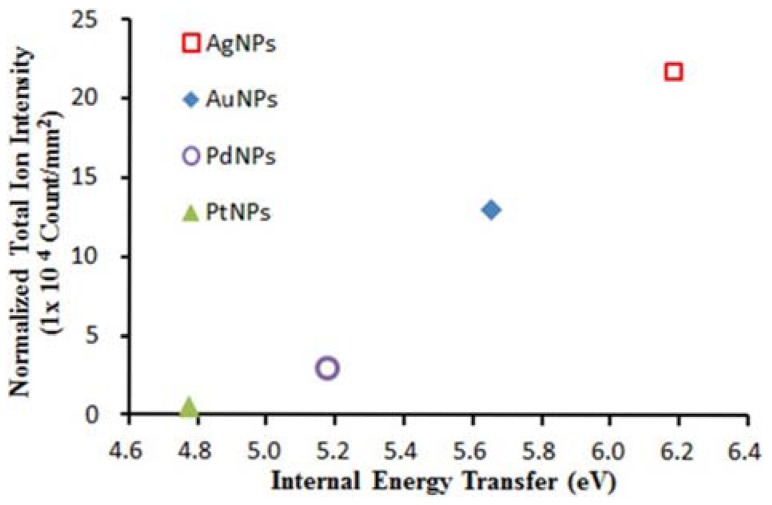
Normalized total ion intensity of benzylpyridinium (BP) ions desorbed from various noble metal NPs *versus* internal-energy transfer at a laser fluence of 52.8 mJ cm^−2^. Reprinted with permission from [58]. Copyright American Chemical Society, 2015.

In this way, the average internal energies of (BP)^+^ desorbed from the different types of noble metal NPs could be determined [58]. All the metal NPs under study exhibited an increase in the total ion intensity on increasing the laser fluence. This trend suggests that a higher laser fluence could enhance the photo-energy deposition which, in turn, promotes the thermal desorption process via laser-induced heating of NPs. The enhanced laser-induced phase transition could also favor the process. Furthermore, in the elegant work by Ng et al. [58], the total ion intensities (*I*_M_ + *I*_F_) were normalized by the total section area of NPs to investigate the effects of the different noble metal NPs on the ion-desorption efficiencies, as shown in Figure 3. The ion desorption efficiency of the different noble metal NPs increased with their corresponding internal-energy transfer (Figure 3), indicating that the thermal-driven desorption (mainly explained by photoabsorption via electronic transitions) might be the key mechanism in the ion-desorption process.

The internal energy of (BP)^+^ desorbed from different noble metal NPs was slightly dependent on the laser fluence and (more importantly), for a given fluence, on the nature of the NPs themselves, strongly suggesting that some difference(s) in physicochemical properties (thermal conductivity, laser-induced heating temperatures, photoabsorption efficiencies, specific heat capacities, densities - see Table 1 [58]) among the metal NPs could govern the extent of internal-energy transfer.

In a thermal-driven process, it appears that a low thermal conductivity could be the most influential factor leading to heat confinement and high ion-desorption efficiency; indeed, thermal conductivity of NPs is generally lower than their bulk counterparts [58]. However, it was demonstrated that the correlation of internal-energy transfer and ion-desorption efficiencies with the thermal conductivities did not follow a specific trend [58]. On the other hand, NP heating and restructuring upon laser irradiation can be promoted by photoabsorption via electronic inter/intraband transitions. The dielectric permittivity of the metal (ε) allows calculation of *Q*_abs_, which, in turn, can be used to finally estimate the laser-induced heating temperature at a given laser fluence. Data in Table 1 indicate that the higher the *Q*_abs_, the higher the temperature reached by NPs that follows the order Ag > Au > Pd > Pt, similar to that found for ion desorption efficiency and internal energy transfer (Figure 3). When NPs with diameters lower than the heat diffusion length are considered, a homogeneous heating of the particles and reduced laser fluence threshold are achieved because of heat diffusion during the laser pulse [68]. Such behavior, initially proposed for metal NP suspensions in glycerol, was also assumed for nanostructures co-deposited with analyte [27]. According to this model, the peak temperature increase, Δ*T*, is inversely proportional to the nanoparticle diameter [9,68] according to Equation (2) (valid for a complete absorption of the laser radiation).

Δ*T* = *H*/ρ*dc* (2)

**Figure 4:**
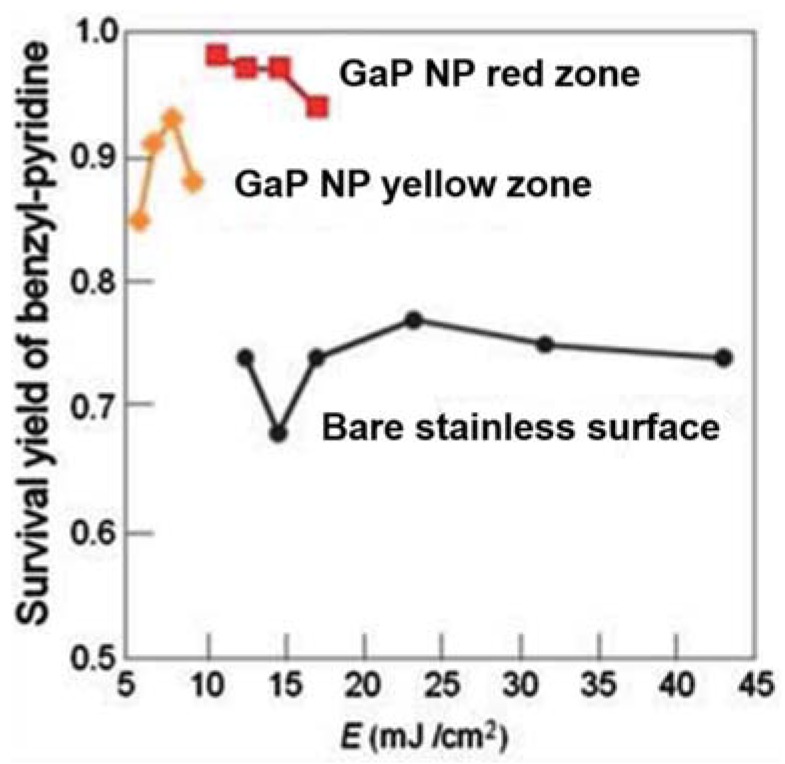
The results of survival yield measurement of various SALDI-MS substrates. (a) GaP NP substrate: red zone (NP size 200–400 nm), yellow zone (NP size 100–200 nm), and bare stainless surface. Adapted from [39] with permission of the Royal Society of Chemistry.

In Equation (2), *H* is the laser fluence, ρ the density, *d* the diameter, and *c* the heat capacity of material. Similar argumentation can explain, for instance, the reduced laser threshold (see Figure 4) needed to desorb 4-chlorobenzylpyridinium (BP-Cl) ions using yellow GaP nanoparticles of size smaller than red GaP NPs [39].

A further interesting example of correlation between ion desorption efficiency (measured by the (BP)^+^ ion intensity) and internal energy transfer (SY measurements), has been provided by Ho-Wai Tang et al. [57] in carbon-based SALDI-MS using multiwalled carbon nanotubes (CNT), buckminsterfullerene (C_60_), nanoporous graphitic carbon (PGC), non-porous graphite particles (G), highly oriented pyrolytic graphite (HOPG), or nanodiamonds (ND). Table 2 summarizes some of the most important properties of these materials that might influence the SALDI behavior.

**Figure 5:**
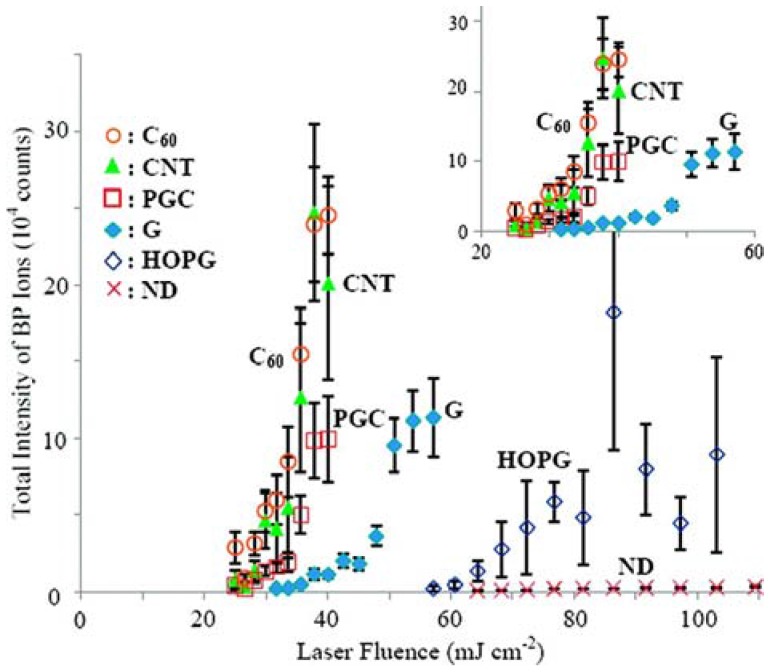
Effect of laser fluence on the total intensity of BP ions desorbed from various carbon substrates. The total ion intensities are the summation of intensities of BP ion at *m*/*z* 170 and the fragment ion at *m*/*z* 91. Adapted with permission from [57]. Copyright American Chemical Society, 2009.

The correlation of total intensity of BP ions with laser fluence for different carbonaceous surfaces is presented in Figure 5 [57].

Remarkable differences were found in terms of threshold laser fluence (CNT ∼ C_60_ < PGC < G < HOPG < ND) and LDI efficiency (CNT ∼ C_60_ > PGC > G > HOPG > ND). The low threshold values reported for CNT, C_60_, and PGC can be ascribed to their reduced size and large surface area and porosity, as compared for example to G and HOPG. In contrast, nanodiamonds with high surface area and good UV absorption did not show comparable performances, indicating that other factors contribute to the process. It was shown that both the desorption efficiency and the extent of internal energy transfer were found dependent on the type and size of the carbon substrates. However, the ion intensity of BP exhibited a trend which was opposite to the extent of internal energy transfer, thus suggesting that increasing the extent of internal energy transfer does not necessarily enhance the ion desorption efficiency.

These findings cannot be fully explained by a thermal desorption mechanism (see the order of thermal conductivity, C_60_ < ND < CNT < G, reported in Table 2), and a cooperating non-thermal desorption mechanism has been proposed. Indeed, the morphological change of the substrates after laser irradiation and the initial velocities of desorbed BP ions (comparable to that of MALDI process mainly involving a phase transition/explosion process) suggested that phase transition/destruction of the substrate was involved in the desorption process. In this respect, other factors could contribute in enhancing the ion desorption efficiency such as the weaker bonding/interaction and/or lower melting point of the substrate (see the order C_60_ < CNT < G < ND reported in Table 2). Charge-transfer process (i.e., electron transfer from carbon substrate to the BP ions, thus suppressing the ion intensity) could be reasonably excluded since the order of ionization energies of the different substrates (Table 2) was found uncorrelated to the observed order of intensities of BP ions.

The importance of phase transition as well as of surface restructuring was highlighted also in other works relevant to metal-based [53,58,60,62,64,69] and silicon-based [22,24,54] materials.

**Figure 6:**
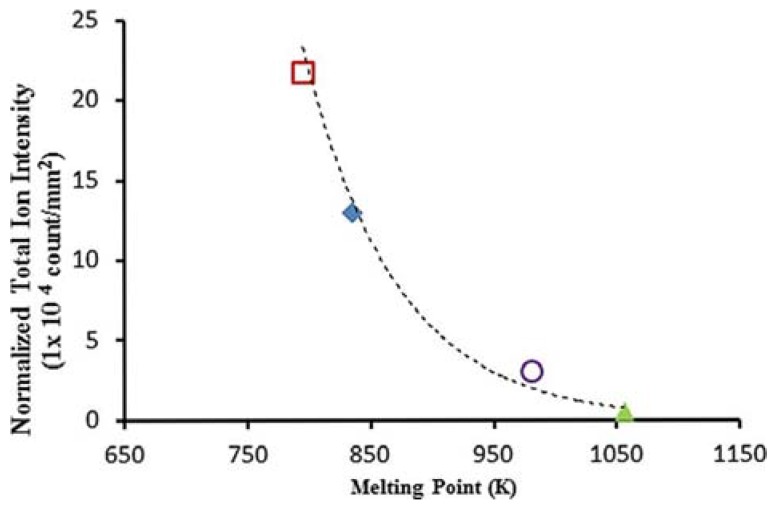
Correlation of normalized total ion intensity of benzylpyridinium (BP) ions desorbed from various noble metal NPs at the laser fluence of 52.8 mJ cm^−2^ with their corresponding melting points. Red Ag, blue Au, purple Pd, green Pt NPs. Reprinted with permission from [58]. Copyright American Chemical Society, 2015.

The melting point of metal NPs should be also taken into account as it could differ remarkably from their bulk counterparts [58]. In fact, NPs showing lower melting points exhibit higher total ion intensities, as rationalized by phase transition thermodynamics (Figure 6) [58].

**Figure 7:**
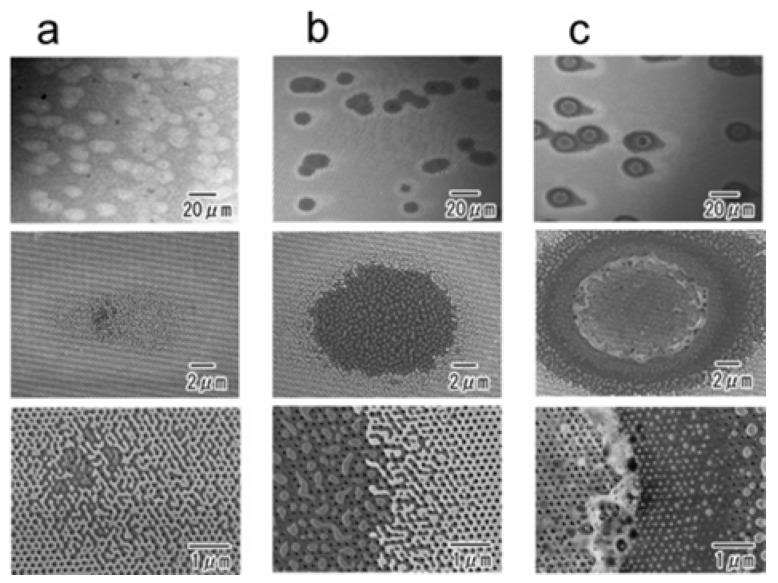
SEM images of laser-irradiated spots. The geometry was 200/100/500 (period/diameter/depth of porous alumina in nm) with 50 nm surface Pt. The levels of applied laser power were ~22 μJ/pulse (**a**), ~28 μJ/pulse (**b**), and ~36 μJ/pulse (**c**). Reprinted with permission from [69]. Copyright American Chemical Society, 2007.

It was observed that a low melting point promoted ion desorption efficiency, though generally resulting in a “harder” process. In fact, AgNPs and AuNPs are more prone to give rise to metal cluster ions [27,58]. Implications of metal surface melting were clearly invoked in SALDI of biomolecules where the process is complicated by analyte charging process (e.g., protonation and/or alkali metal cationization). Wada et al. used platinum- or gold-coated porous alumina with submicrometer structures for SALDI MS of angiotensin I, trypsinogen and 15-kDa ribonuclease B [69]. The melting temperature of bulk Pt is 2041 K, but size effects result in melting at much lower temperatures. Indeed, melting of surface platinum upon laser irradiation at the fluence sufficient to generate peptide ions was confirmed by scanning electron microscopy (SEM) (Figure 7) [69].

Three-dimensional platinum nanoflowers (Pt Nfs) on a scratched silicon substrate were synthesized by Kawasaki et al. [64], and showed to possess excellent SALDI activity in terms of the efficient generation of protonated molecular ions for peptides and various analytes, including phospholipids, carbohydrates, and synthetic polymers. Here, proton transfer from the Si–OH groups to the analyte molecules is enhanced, due to an increased acidity of sylanols because of the electron-withdrawing nature of the Pt Nfs; protonated analytes are finally thermally desorbed from the surface. SEM imaging of the Pt Nfs structure after laser irradiation near the threshold (50 mJ cm^−2^) for peptide detection, demonstrated that there were minor morphological changes, indicating that surface reconstruction/destruction of Pt Nfs plays only a minor role.

The effect of various phase transition stages on SALDI ion desorption efficiency and energy transfer extent (BP as chemical thermometer) were investigated by Lai et al. [62]. Molecular dynamics was used to simulate phase transition (from melting, vaporization, to phase explosion) of AuNPs upon laser irradiation employing (for the first time) a wide range of fluencies (21.3 mJ cm^−2^ to 125.9 mJ cm^−2^). In this study, AuNPs phase explosion (threshold temperature of 5800 K) was found to have the most significant effect on enhancing the ion desorption efficiency and lowering the extent of energy transfer (i.e., increase in SYs). Vaporization played only a limited effect on the ion desorption efficiency, while the effect of melting was in practice not appreciable. Enhancement of the ion desorption efficiency during phase explosion was attributed to the weaker binding between the BP ions and the rapidly ablated Au atoms. The increase in the SYs observed at laser fluence values at which a simultaneous sudden increase in the total ion intensity also occurred, was explained invoking cooling of BP ions during the adiabatic expansion of the ablation plume.

It was also shown that surface functionalization of nanomaterials influences the LDI performance in terms of process “softness” and/or internal energy transfer. For instance, the internal energy deposition in (silylated) silicon nanoparticle-assisted laser desorption/ionization (SPALDI) was evaluated by Dagan et al. [61], and compared to LDI from underivatized SiNPs and MALDI-CHCA (Table 3). Five BP derivatives were used as thermometer molecules and an internal energy threshold ranging from 2.8 eV to 3.7 eV was estimated. This is, once again, significantly lower than that reported for DIOS, which requires much higher laser fluence thresholds [70]. Contrary to what observed for SiNWs [54], the SYs in SPALDI was found to gradually decrease with increasing laser fluence. Unmodified SiNPs exhibited the highest analyte internal energy compared to the silylated ones that, in turn, acted differently depending on the nature of the derivatizing silane used. Indeed, it was found that the lower the length of the modifier’s alkyl chain (C10, C6, C3), the higher was the internal energy deposited (Table 3), suggesting a role of the modifier itself as a moderator in the energy dissipation and relaxation process. Keeping in mind that the internal energy is inversely correlated to SY, reduced ion fragmentation occurs on functionalized SiNPs as compared to untreated ones [61]. It is worth of note that for the BP model compounds, SPALDI with C10 silylated SiNPs produces SYs similar or higher (at the threshold fluence range) than conventional MALDI-CHCA [61].

Note, however, that the BP model compounds are pre-charged ions, which is usually not the case in conventional MALDI, where e.g., proton transfer may also occur in the gas phase, significantly affecting the internal energy of the formed ions.

**Figure 8:**
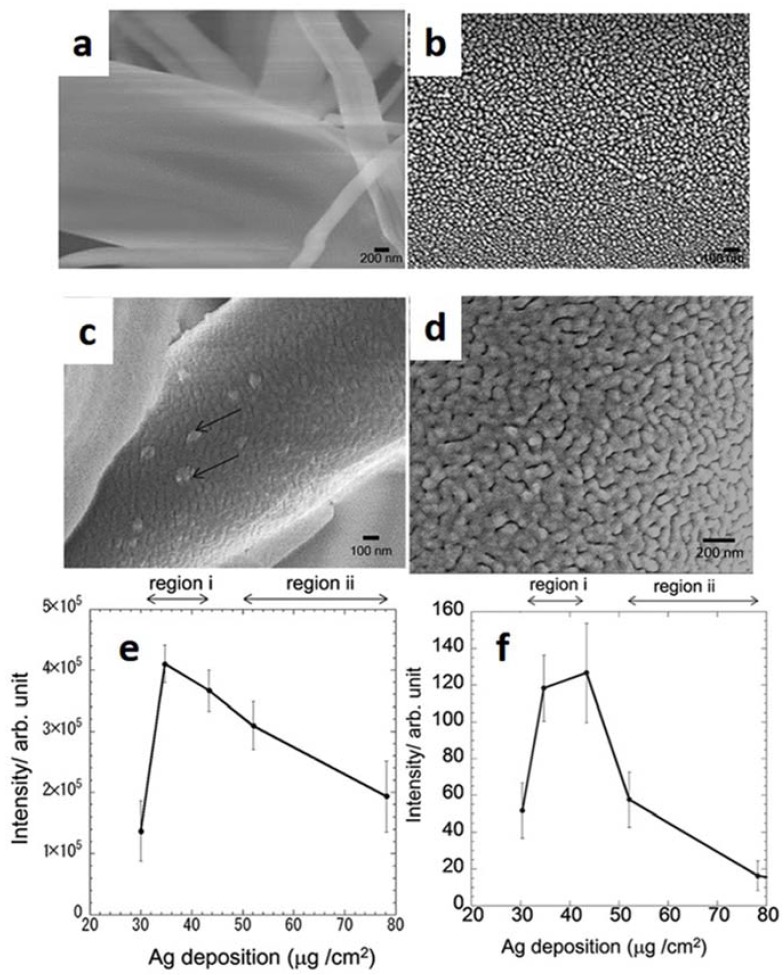
SEM images of the Ag NP-GF substrates with different amounts of deposited Ag of (**a**,**b**) 35 μg/cm^2^; (**c**) 52 μg/cm^2^ (arrows denote bulk silver islands), and (**d**) 78 μg/cm^2^. Effect of the amount of deposited Ag on: (**e**) the SERS peak intensity (1066 cm^−1^), and (**f**) SALDI peak intensity (*m/z* = 124) of 4-aminothiophenol (4-ATP). Region (i) 30 μg/cm^2^–43 μg/cm^2^, region (ii) 43 μg/cm^2^–78 μg/cm^2^. Adapted from [48] with permission of the Royal Society of Chemistry.

LDI efficiency may be influenced by uniform distribution of polymer-immobilized AuNPs, as demonstrated by Tarui et al. [34], who showed how controlled aggregation of NPs allowed operating at reduced laser fluencies, thus decreasing background signals from Au cluster ions. In this case, a remarkable temperature rise was explained by the amplified heating effect due to adjacent nanoparticles, present in the aggregates, which heat each other. The importance of inter-particle distance was addressed by Kurita et al. [48], who reported how AgNPs of 20 nm–50 nm size sputtered onto glass fibers (Ag NP-GF substrates) were particularly efficient as both SERS and SALDI substrates when distributed in a closely-packed structure with inter-particle gaps < 10 nm (Figure 8a,b). Increasing the amount of deposited silver led to interconnected islands of larger size (Figure 8c,d), thus worsening overall analytical performance. In fact, a similar trend on SERS and SALDI signals was found as a function of deposited Ag (Figure 8e,f).

Despite NP properties and composition, analyte binding to NP surface may play a role as high affinity could limit analyte ion desorption, thus reducing LDI efficiency [58]. On the other hand, chemical interactions between nanophases and analyte molecules may be essential for promoting selective and highly sensitive LDI detection [71]. Yagnik et al. [71] reported that LDI efficiency of non-functionalized (metal or metal oxide) NPs can be mainly explained by a thermal desorption model, though other aspects concerning analyte-NP interactions and surface adsorption may play a role, especially for metal NPs.

## 3. Other Aspects Influencing Desorption/Ionization Mechanisms

Besides the importance of NP size [59,72] and composition [60], discussed in the previous section, other factors have been reported to influence the LDI process. Since the early appearance of nanostructures for MS applications, it was shown that surface plasmon (SP) excitation could enhance both the electromagnetic field and the charge transfer between the NP and the adsorbate molecules [73], thus affecting both desorption and ionization phenomena [59,74,75]. Such correlation was deeply studied for gold nanostructures, demonstrating that a visible laser at 532 nm, which can be resonantly absorbed by AuNPs (SP band falling in the 500 nm–600 nm range), positively affects analyte detection [74,75,76,77], provided that no aggregation phenomena occur. Interestingly, AuNP absorption associated to broad interband in the UV region may also promote the LDI process through thermionic emission of electrons by interband excitation when a UV laser is used [59]. A similar behavior was also found for AgNPs (20 nm–50 nm) in a closely-spaced assembly [48]. A complete overview on interactions between pulsed lasers and gold nanoparticles can be found elsewhere [78]. Similarly, other optical features (e.g., reflectivity) affect the LDI-MS performance. Pd nanostructured materials, such as PdNPs deposited onto porous silicon, showed low reflectivity, which corresponded to good LDI performance [79]. Such parameter has also been considered for Si-based materials in several works [76,80,81,82].

**Figure 9:**
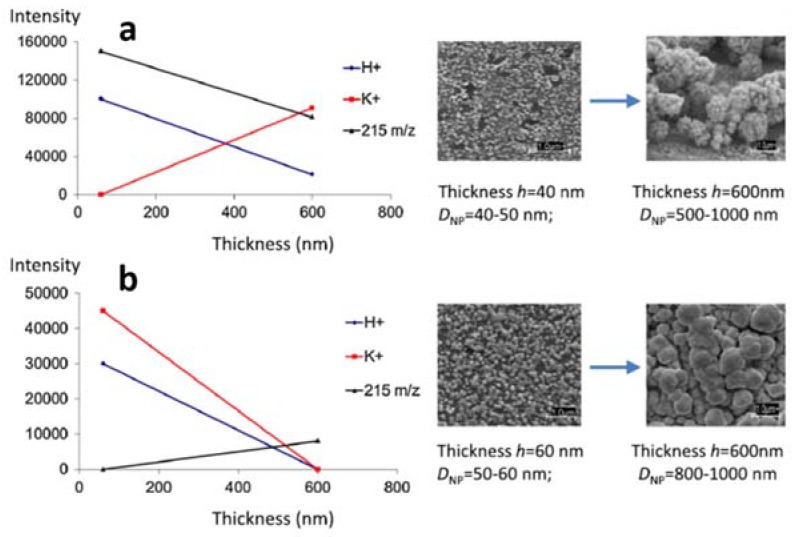
Dependence of (M + H)^+^, (M + K)^+^ and *m/z* 215 product ion (deriving from sulfamethazine dissociation) intensities on thickness, morphology and diameter for (**a**) Pd nanoflower coatings on porous silicon, and (**b**) Pd-NPs on polished steel. (10 ng/spot sulfamethazine; laser fluence, 40%). Reproduced with permission from [79]. Copyright 2014 John Wiley and Sons.

Regarding the morphological properties of nanostructures, two main aspects received attention, namely shape and surface roughness [54,64,69,79,83,84,85,86]. In particular, the role of different shapes (spheres, rods, and stars) of AuNPs [75] as well as the role of morphology of Pd [79] (Figure 9) and Pt nanostructured materials [87,88] were investigated. Several groups also carried out similar works on Si-based materials [81,82,89]. It is generally accepted that sub-micrometer roughness (or porosity) and high surface area are essential to achieve acceptable ion desorption efficiencies [80,90,91].

It was shown that Pd film thickness (*h*) was an important feature, in combination with the nature of the support (porous Si *vs.* steel) (Figure 9) [79]. In the case of porous Si (Figure 9a), increasing *h* caused the increase of nanoflowers diameter from D_NP_ ~ 50 nm to 500 nm–1000 nm which, in turn, resulted in a reduced formation of protonated molecular ion and less probable fragmentation (lower intensity of 215 *m/z* peak due to analyte dissociation). An opposite behavior was found for PdNPs supported on steel (Figure 9b), indicating degradation of molecular species and gradual loss of SALDI activity (absence of (M + H)^+^, (M + K)^+^ signals).

Despite the chemical composition of nanophases, surface modification was often applied to enhance LDI efficiency [92], while limiting excessive fragmentation [67]. In fact, such coatings can facilitate energy dissipation, thus promoting “softer” ionization [61,79,84,85], or can assist analyte ionization when a conventional matrix is covalently attached to NP surface [35,93]. Moreover, specific modification of NPs can lead to enrichment and selective detection [94,95,96,97,98], as in the case of aptamer-modified gold nanofilms applied to the analysis of circulating tumor cells [99]. Nonetheless, it is worth mentioning that a general consensus was reached on the preferential use of “bare” nanostructures as compared to “stabilized” ones in LDI applications [58,100,101,102].

On the other hand, the role of the analyte reactivity [71] towards nanostructured surfaces (see e.g., Ag and Au for thiol groups, Ag for double bonds [103], TiO_2_ for phosphate) as well as intrinsic properties (e.g., proton affinity [88,104]) should be considered as well. The role of adsorbed water was also assessed on porous graphite and silicon surfaces [55], suggesting that LDI efficiency was significantly reduced after removal of physisorbed/chemisorbed water or hydroxylated silicon terminal groups [79].

## 4. Promising Methods to Study Laser Radiation-Nanophase Interactions

The investigation of the nature of interactions arising when pulsed laser radiation hits a nanostructure is an almost unexplored field in LDI-MS. More frequently, microscopy techniques (e.g., atomic force microscopy—AFM, scanning electron microscopy—SEM) have been applied for the characterization of LDI nanostructured substrates, before and after laser irradiation [39,54,69,77,79,105,106]. However, “in situ” techniques as well as theoretical modelling would be important to study the process. For example, Pustovalov [107] took into account the temperature dependences of optical and thermo-physical parameters relevant to different NPs and surrounding environments (e.g., water, liquids) to model the laser pulse-NP interactions. Computational modelling for plasmonic gold nanoparticles was also proposed by Hashimoto and colleagues [108], as reviewed in [78].

In principle, the use of nanophases as LDI promoters for MS detection could be combined with an emerging technique based on Laser Induced Breakdown Spectroscopy (LIBS) enhanced by the addition of NPs [109]. Conventional LIBS allows investigating sample chemical composition by the analysis of element emission spectra [110]. In Nanoparticle-Enhanced (NE) LIBS, thanks to the peculiar features of NP-laser radiation interactions, the breakdown threshold for solid targets [111,112] as well as for biological samples [113] could be significantly reduced allowing for sensitive detection. In this respect, a picture of the analyzed sample, from the spectroscopic and MS points of view, may be simultaneously acquired shading light on mechanistic and qualitative aspects of nanomaterial-based LDI approaches. As a result, the application of time-resolved spectroscopies and/or imaging techniques may be helpful to unravel LDI mechanisms involving nanoparticles interacting with pulsed lasers [114].

## 5. Conclusions

An overview about the general aspects of desorption/ionization mechanisms concerning nanostructured materials used in matrix-free LDI-MS has been provided in this work. Despite the remarkable number of reported SALDI-MS applications, the number of theoretical studies in this field is pretty low. More recently, such topics have been addressed focusing on the close correlation between nanostructure physicochemical parameters and internal energy transfer using benzylpyridinium salts as model compounds. Undoubtedly, heat-confinement, melting, and photoabsorption, which depend on the chemical nature and size of the nanomaterials, are the main factors influencing the LDI process. However, other features (e.g., roughness, hydrophobicity, capping agents, analyte properties) might play a role complicating the process understanding. To this aim, additional characterizations should be carried out, such as computational modelling of laser radiation-NP interactions, morphological and spectroscopic investigations of the nanomaterials.

## Figures and Tables

**Table 1:** Specific Heat Capacities, Densities, Dielectric Permittivities (Real and Imaginary Parts, ε’ and ε”), Photoabsorption Efficiencies (*Q*_abs_), and Laser-Induced Heating Temperatures of AgNPs, AuNPs, PdNPs, and PtNPs. Laser irradiation at 355 nm, Fluence = 52.8 mJ cm^−2^. For more details about data herein reported, please refer to [58]. Adapted with permission from [58]. Copyright American Chemical Society, 2015.

Metal NPs	Specific Heat Capacity (J·g^−1^·K^−1^)	Density at 298 K (g·cm−^3^)	ε′	ε″	(Q_abs_)	Laser-Induced Heating Temperature (K)
**AgNPs**	0.235	10.5	−2.04	0.28	0.18525	18,836
**AuNPs**	0.129	19.3	−1.24	5.60	0.00551	943
**PdNPs**	0.244	12.0	−5.54	6.53	0.00365	569
**PtNPs**	0.133	21.5	−3.96	8.18	0.00353	542

**Table 2:** Selected Physical and Physicochemical Properties of Multiwalled Carbon Nanotubes (CNT), Buckminsterfullerene (C_60_), Graphite (G), and Nanodiamonds (ND). For more details about data herein reported, please refer to [57]. Adapted with permission from [57]. Copyright American Chemical Society, 2009.

	CNT	C_60_	G	ND
**Specific heat capacity (J·g^−1^ K^−1^) at ~300 K**	0.45	0.7	−0.71	0.68
**Thermal conductivity (Wcm^−1^K^−1^) at ~300 K**	0.25	0.004	19.5	0.12
**Melting point (K)**	1600–3200	1000 (sublimation)	3800–4762	~5000 (at 8.5 GPa)
**Laser Fluence Threshold for damaging carbon substrates by using Nd:YAG laser (λ = 620 nm), pulse width = 90 fs) (J cm^−2^)**	Not available	Not available	0.13	0.63
**Ionization potential (eV)**	5.3	7.6	4.39	6.9–8.07

**Table 3:** Critical energy and calculated lowest internal energy of BP ions generated by SPALDI with chemically modified and untreated Si nanoparticles, and by MALDI with CHCA. 70 ns was used as the reaction time in the calculation. The results represent the energy for a laser fluence that induced minimal, yet measurable fragmentation. 4-Cl BP = 4-chloro benzylpyridinium, 2-Me BP = 2-methyl benzylpyridinium, 4-Me BP = 4-methyl benzylpyridinium, 3-MeO BP = 3-methoxy benzylpyridinium, 4-MeO BP = 4-methoxy benzylpyridinium. For more details about data herein reported, please refer to [61]. Adapted with permission from [61] Copyright Elsevier B.V, 2009.

	Critical Energy, *E*_0_ (eV)	Calculated Lowest Internal Energy of BP Ions (eV)				
		C10 Modified Si	C6 Modified Si	C3 Modified Si	Untreated Si	CHCA
**4-CL BP**	1.73	3.5	-		4.5	3.7
**2-ME BP**	1.64	3.4	-		4.2	3.6
**4-ME BP**	1.6	3.4	-		4.1	3.0
**3-MEO BP**	1.68	3.7	-		4.8	4.3
**4-MEO BP**	1.3	2.8	3.0	3.5	3.9	3.2
